# Gleanings from Our Exchanges

**Published:** 1872-06-01

**Authors:** 


### Gleanings from Our Exchanges.

On the Early History of Calculous Disease, and the Treatment Best Adapted for its Prevention.—Sir Henry Thompson publishes a lecture on this subject, of which we give an abstract. He commences by saying that "all calculi are of local or of constitutional origin. By 'local,' I mean formed by disease in the bladder, and not depending upon any constitutional conditions; by 'constitutional,' I mean formed by some vice in the system." The large majority of stones are of constitutional origin. Of these, by far the largest proportion have uric acid for their bases; a few have bases of oxalate of lime; and still fewer have phosphate of lime for bases: therefore, the practical problem is to prevent the formation of uric acid calculus.

He has found that the case almost invariably is that either calculus, or gravel, which is the same thing, or gout, and most commonly the latter, has existed in the preceding generation.

The first sign of this condition is the persistent, or, at all events, the frequent, deposition of a pinkish matter at the bottom of the vessel on cooling; or, the urine may be merely cloudy when cold. This is only a deposition of salts from a warm solution on cooling, which will be redissolved on warming the liquid again.

Such a deposit may occur occasionally in the most healthy person, in consequence of some error of diet. It is only when it is of frequent occurrence that it is of any consequence. But if the patient, without any errr of diet, habitually passes this kind of urine; if in time there is a manifest deposit of uric acid in the bottom of the vessel, looking like particles of cayenne pepper, then there is no doubt about the tendency to the production of uric acid. This deposit consists of crystals of uric acid. They may be passed almost daily, or at intervals; and at such times the patient may experience pains in the back, and great discomfort, and may then be said to have an attack of sand or gravel.

This condition is produced by the same cause as gout, and may alternate with it.

The treatment that is generally employed for these cases is the administration of alkalies when acids are deposited; if alkalies are present, acids are given. Such treatment may give temporary relief, but will not permanently benefit the patient.

At this point he makes a digression, and speaks of the pathology of the disease. He considers that the origin of gouty symptoms, as well as superabundant uric acid deposits in the urine, is due to defective assimilation of organs associated with or forming the primæ viæ. At the bottom of all this there often lies what is commonly understood by inactivity or torpidity of the liver, whatever that may be. The *rationale* of the trouble he considers to be that the liver, or some allied organ, does not do its duty ; consequently, more work is thrown upon the kidneys; and thus the solid matters of the urine, or rather some of its ordinary constituents, are augmented.

Hence, the treatment which he recommends is "to employ such agents as will stimulate the primæ viæ without depressing vital power." A powerful agent for this purpose is mercury; and relief for such symptoms may be obtained by occasional small doses of that drug. But the means which he particularly recommends on account of their superior safety and efficiency, are mineral waters which contain large proportions of sulphate of soda. For this purpose the artificially prepared waters will not do. The natural waters must be used, or the result will disappoint our expectations.

The medicine which he considers next in efficiency, when the mineral waters can not be obtained, is Glauber's salts.

In regard to diet, he prohibits, or allows in very small quantities, alcohol, saccharine, and fatty matters.—*Lancet.*

The Lung Test.—M. Poucet showed, at a meeting of the Lyons *Societe des Sciences Medicales*, the lungs of a fœtus prematurely born. The child had cried, breathed, and lived an extra-uterine life of ten hours; but the lungs sank completely in water, as if no respiration had taken place. Other cases of the same kind have been related by other observers, showing the insecurity of the lung test in forensic medicine.—*Lancet.*

Benzine in Whooping-Cough.— Dr. Rottard says that benzine internally administered in from 10 to 20 drop doses, is the best remedy yet mentioned in whooping-cough. It may be used either alone or in connection with aspirations of the vapor.

Treatment of Enurosis.— Sponging the lower part of the spine before going to bed is said to have greatly benefited this troublesome complaint in children, when all other treatment had failed.

